# 2-(1*H*-1,2,3-Benzotriazol-1-yl)-1-*m*-toluoylethyl 2,4-dichloro­benzoate

**DOI:** 10.1107/S1600536808011963

**Published:** 2008-04-30

**Authors:** Wu-Lan Zeng

**Affiliations:** a MicroScale Science Institute, Department of Chemistry and Chemical Engineering, Weifang University, Weifang 261061, People’s Republic of China

## Abstract

In the title compound, C_23_H_17_Cl_2_N_3_O_3_, the dihedral angles between the mean planes of the benzotriazole system and the methyl- and dichloro-substituted benzene rings are 47.72 (1) and 13.06 (1)°, respectively. In the crystal structure, inter­molecular C—H⋯O and C—H⋯π inter­actions help to consolidate the packing.

## Related literature

For background, see Chen & Wu (2005 ▶). For reference structural data, see: Allen *et al.* (1987 ▶).
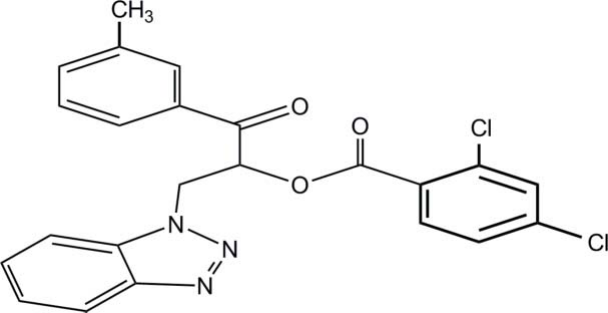


## Experimental

### 

#### Crystal data

C_23_H_17_Cl_2_N_3_O_3_
*M* *_r_* = 454.30
Monoclinic, 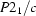

*a* = 9.3395 (19) Å
*b* = 9.3065 (19) Å
*c* = 23.538 (5) Å
β = 92.10 (3)°
*V* = 2044.5 (7) Å^3^
*Z* = 4
Mo *K*α radiation
μ = 0.35 mm^−1^
*T* = 113 (2) K
0.18 × 0.16 × 0.08 mm

#### Data collection

Bruker SMART CCD diffractometer
Absorption correction: multi-scan (*SADABS*; Bruker, 1997 ▶) *T*_min_ = 0.940, *T*_max_ = 0.973
11260 measured reflections
3585 independent reflections
3138 reflections with *I* > 2σ(*I*)
*R*_int_ = 0.031

#### Refinement

*R*[*F* ^2^ > 2σ(*F* ^2^)] = 0.033
*wR*(*F* ^2^) = 0.091
*S* = 1.10
3585 reflections
281 parameters
H-atom parameters constrained
Δρ_max_ = 0.24 e Å^−3^
Δρ_min_ = −0.30 e Å^−3^

### 

Data collection: *SMART* (Bruker, 1997 ▶); cell refinement: *SAINT* (Bruker, 1997 ▶); data reduction: *SAINT*; program(s) used to solve structure: *SHELXS97* (Sheldrick, 2008 ▶); program(s) used to refine structure: *SHELXL97* (Sheldrick, 2008 ▶); molecular graphics: *SHELXTL* (Sheldrick, 2008 ▶); software used to prepare material for publication: *SHELXTL*.

## Supplementary Material

Crystal structure: contains datablocks global, I. DOI: 10.1107/S1600536808011963/hb2721sup1.cif

Structure factors: contains datablocks I. DOI: 10.1107/S1600536808011963/hb2721Isup2.hkl

Additional supplementary materials:  crystallographic information; 3D view; checkCIF report

## Figures and Tables

**Table 1:** Hydrogen-bond geometry (Å, °)

*D*—H⋯*A*	*D*—H	H⋯*A*	*D*⋯*A*	*D*—H⋯*A*
C15—H15⋯O1^i^	0.93	2.35	3.263 (2)	168
C4—H4⋯*Cg*1^ii^	0.93	2.86	3.4645 (19)	124

Symmetry codes: (i) 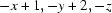; (ii) 
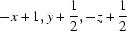
. *Cg*1 is the centroid of the C18–C23 ring.

**Fig. 1.**
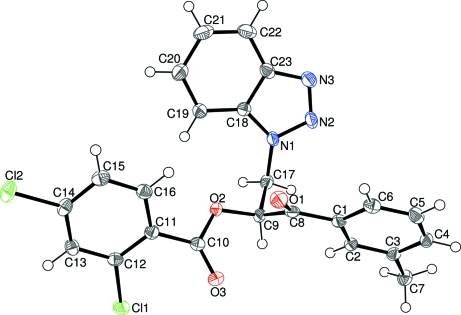
The molecular structure of (I), drawn with 50% probability ellipsoids (arbitrary spheres for the H atoms).

## supplementary crystallographic information

### Comment

1*H*-Benzotriazole and its derivatives exhibit a broad spectrum of pharmacological activities such as antifungal, antitumor and antineoplastic activities (Chen & Wu, 2005). We report here the synthesis and structure of the title compound, (I) (Fig. 1), as part of our ongoing studies on new benzotriazole compounds with higher bioactivity.

The molecule of (I) is chiral.  In the arbitrarily chosen asymmetric molecule, C9 has S configuration, but crystal symmetry generates a racemic mixture. Otherwise, all the bond lengths and angles in (I) are within their normal ranges (Allen *et al.*, 1987). The benzotriazole ring system is essentially planar, with a dihedral angle of 2.2 (8)° between the triazole ring (atoms N1—N3/C18/C23) and the C18—C23 benzene ring. The dihedral angles between the mean planes of the benzotriazole system and the C1—C6 and C11—C15 aromatic rings are 47.72 (1)° and 13.06 (1)°, respectively. The dihedral angle between rings C1—C6 and C11—C15 is 35.26 (2)°.

In the crystal, intermolecular C-H···O and C-H···π interactions (Table 1) help to consolidate the packing.

### Experimental

Bromine (3.2 g, 0.02 mol) was added dropwise to a solution of 3-(1*H*-benzo[*d*][1,2,3]triazol-1-yl)-1-*m*-tolylpropan-1-one (5.30 g, 0.02 mol) and sodium acetate (1.6 g, 0.02 mol) in acetic acid (50 ml). The reaction proceeded for 7 h. Water (50 ml) and chloroform (20 ml) were then added. The organic layer was washed successively with saturated sodium bicarbonate solution and brine, dried over anhydrous magnesium sulfate and the chloroform solution filtered. It was cooled with ice-water, and then an acetone solution (10 ml) of 2-chlorobenzoic acid (3.8 g, 0.02 mol) and triethylamine (2.8 ml) was added. The mixture was stirred with ice-water for 6 h. The solution was then filtered and concentrated. Colourless slabs of (I) were obtained by slow evaporation of ethanol solution at room temperature after one week.

### Refinement

The H atoms were geometrically placed (C—H = 0.93–0.97 Å), and refined as riding with *U*_iso_(H) = 1.2*U*_eq_(C) or 1.5*U*_eq_(methyl C).

### Crystal data

**Table:**

C_23_H_17_Cl_2_N_3_O_3_	*F*_000_ = 936
*M**_r_* = 454.30	*D*_x_ = 1.476 Mg m^−^^3^
Monoclinic, *P*2_1_/*c*	Mo *K*α radiation λ = 0.71073 Å
Hall symbol: -P 2ybc	Cell parameters from 5663 reflections
*a* = 9.3395 (19) Å	θ = 1.7–27.9º
*b* = 9.3065 (19) Å	µ = 0.35 mm^−^^1^
*c* = 23.538 (5) Å	*T* = 113 (2) K
β = 92.10 (3)º	Slab, colourless
*V* = 2044.5 (7) Å^3^	0.18 × 0.16 × 0.08 mm
*Z* = 4	

### Data collection

**Table:**

Bruker SMART CCD diffractometer	3585 independent reflections
Radiation source: X-ray tube	3138 reflections with *I* > 2σ(*I*)
Monochromator: graphite	*R*_int_ = 0.031
*T* = 113(2) K	θ_max_ = 25.0º
ω scans	θ_min_ = 2.4º
Absorption correction: multi-scan(SADABS; Bruker, 1997)	*h* = −11→10
*T*_min_ = 0.940, *T*_max_ = 0.973	*k* = −11→10
11260 measured reflections	*l* = −27→20

### Refinement

**Table:**

Refinement on *F*^2^	Secondary atom site location: difference Fourier map
Least-squares matrix: full	Hydrogen site location: inferred from neighbouring sites
*R*[*F*^2^ > 2σ(*F*^2^)] = 0.033	H-atom parameters constrained
*wR*(*F*^2^) = 0.091	*w* = 1/[σ^2^(*F*_o_^2^) + (0.0569*P*)^2^ + 0.0675*P*] where *P* = (*F*_o_^2^ + 2*F*_c_^2^)/3
*S* = 1.10	(Δ/σ)_max_ = 0.001
3585 reflections	Δρ_max_ = 0.24 e Å^−^^3^
281 parameters	Δρ_min_ = −0.30 e Å^−^^3^
Primary atom site location: structure-invariant direct methods	Extinction correction: none

### Special details

**Table:**

Geometry. All e.s.d.'s (except the e.s.d. in the dihedral angle between two l.s. planes) are estimated using the full covariance matrix. The cell e.s.d.'s are taken into account individually in the estimation of e.s.d.'s in distances, angles and torsion angles; correlations between e.s.d.'s in cell parameters are only used when they are defined by crystal symmetry. An approximate (isotropic) treatment of cell e.s.d.'s is used for estimating e.s.d.'s involving l.s. planes.
Refinement. Refinement of *F*^2^ against ALL reflections. The weighted *R*-factor *wR* and goodness of fit *S* are based on *F*^2^, conventional *R*-factors *R* are based on *F*, with *F* set to zero for negative *F*^2^. The threshold expression of *F*^2^ > σ(*F*^2^) is used only for calculating *R*-factors(gt) *etc*. and is not relevant to the choice of reflections for refinement. *R*-factors based on *F*^2^ are statistically about twice as large as those based on *F*, and *R*- factors based on ALL data will be even larger.

### Fractional atomic coordinates and isotropic or equivalent isotropic displacement parameters (Å^2^)

**Table:**

	*x*	*y*	*z*	*U*_iso_*/*U*_eq_	
Cl1	0.16956 (4)	0.45085 (4)	0.044626 (17)	0.02736 (13)	
Cl2	0.48958 (4)	0.53198 (5)	−0.135572 (17)	0.03077 (14)	
O1	0.28238 (12)	1.07298 (12)	0.10792 (5)	0.0272 (3)	
O2	0.37879 (10)	0.80863 (11)	0.11568 (4)	0.0208 (3)	
O3	0.14534 (11)	0.76130 (12)	0.09646 (5)	0.0309 (3)	
N1	0.58217 (13)	0.98801 (13)	0.18828 (6)	0.0199 (3)	
N2	0.61195 (13)	1.10055 (14)	0.22405 (6)	0.0255 (3)	
N3	0.71818 (13)	1.17383 (14)	0.20467 (6)	0.0279 (3)	
C1	0.22159 (14)	1.10721 (16)	0.20360 (7)	0.0189 (3)	
C2	0.18316 (15)	1.04219 (16)	0.25408 (7)	0.0194 (3)	
H2	0.1983	0.9441	0.2590	0.023*	
C3	0.12259 (14)	1.12125 (16)	0.29721 (7)	0.0209 (3)	
C4	0.10223 (15)	1.26887 (16)	0.28894 (7)	0.0228 (4)	
H4	0.0603	1.3232	0.3170	0.027*	
C5	0.14371 (15)	1.33491 (17)	0.23954 (7)	0.0244 (4)	
H5	0.1320	1.4336	0.2352	0.029*	
C6	0.20216 (15)	1.25563 (16)	0.19677 (7)	0.0231 (4)	
H6	0.2287	1.3004	0.1634	0.028*	
C7	0.08028 (17)	1.05045 (18)	0.35132 (7)	0.0272 (4)	
H7A	−0.0178	1.0198	0.3476	0.041*	
H7B	0.0908	1.1176	0.3822	0.041*	
H7C	0.1407	0.9687	0.3588	0.041*	
C8	0.27983 (15)	1.02509 (16)	0.15583 (7)	0.0197 (3)	
C9	0.34219 (15)	0.87504 (16)	0.16804 (6)	0.0198 (3)	
H9	0.2707	0.8162	0.1867	0.024*	
C10	0.26778 (15)	0.75666 (16)	0.08292 (7)	0.0213 (3)	
C11	0.32137 (15)	0.69822 (16)	0.02907 (7)	0.0194 (3)	
C12	0.28115 (15)	0.56368 (16)	0.00791 (7)	0.0193 (3)	
C13	0.33423 (15)	0.51230 (17)	−0.04221 (6)	0.0212 (4)	
H13	0.3086	0.4214	−0.0555	0.025*	
C14	0.42553 (15)	0.59738 (17)	−0.07222 (7)	0.0221 (3)	
C15	0.46799 (15)	0.73162 (17)	−0.05280 (7)	0.0239 (4)	
H15	0.5301	0.7878	−0.0734	0.029*	
C16	0.41582 (15)	0.77997 (17)	−0.00211 (7)	0.0220 (3)	
H16	0.4445	0.8696	0.0116	0.026*	
C17	0.47953 (15)	0.87985 (16)	0.20522 (7)	0.0210 (3)	
H17A	0.4545	0.8983	0.2442	0.025*	
H17B	0.5251	0.7863	0.2043	0.025*	
C18	0.67506 (14)	0.98839 (16)	0.14484 (7)	0.0188 (3)	
C19	0.69518 (16)	0.89704 (17)	0.09888 (7)	0.0227 (4)	
H19	0.6366	0.8177	0.0919	0.027*	
C20	0.80604 (16)	0.93045 (19)	0.06451 (8)	0.0300 (4)	
H20	0.8227	0.8727	0.0332	0.036*	
C21	0.89552 (17)	1.05053 (19)	0.07554 (8)	0.0342 (4)	
H21	0.9701	1.0692	0.0515	0.041*	
C22	0.87541 (16)	1.13949 (18)	0.12039 (8)	0.0301 (4)	
H22	0.9346	1.2184	0.1273	0.036*	
C23	0.76221 (15)	1.10800 (16)	0.15590 (7)	0.0239 (4)	

### Atomic displacement parameters (Å^2^)

**Table:**

	*U*^11^	*U*^22^	*U*^33^	*U*^12^	*U*^13^	*U*^23^
Cl1	0.0300 (2)	0.0263 (2)	0.0262 (2)	−0.00985 (15)	0.00706 (17)	−0.00106 (17)
Cl2	0.0362 (2)	0.0404 (3)	0.0161 (2)	0.00522 (17)	0.00562 (18)	−0.00085 (17)
O1	0.0329 (6)	0.0272 (6)	0.0216 (7)	−0.0044 (5)	0.0046 (5)	0.0035 (5)
O2	0.0188 (5)	0.0247 (6)	0.0192 (6)	−0.0027 (4)	0.0041 (4)	−0.0069 (5)
O3	0.0210 (6)	0.0373 (7)	0.0351 (8)	−0.0070 (5)	0.0090 (5)	−0.0115 (6)
N1	0.0210 (6)	0.0172 (6)	0.0214 (8)	0.0009 (5)	0.0001 (5)	−0.0044 (5)
N2	0.0260 (7)	0.0209 (7)	0.0294 (8)	0.0027 (5)	−0.0027 (6)	−0.0096 (6)
N3	0.0238 (7)	0.0219 (7)	0.0378 (9)	−0.0003 (5)	−0.0015 (6)	−0.0053 (7)
C1	0.0153 (7)	0.0201 (8)	0.0212 (9)	−0.0003 (6)	−0.0013 (6)	−0.0009 (7)
C2	0.0160 (7)	0.0183 (8)	0.0241 (9)	0.0002 (5)	0.0013 (6)	−0.0017 (7)
C3	0.0144 (7)	0.0257 (8)	0.0224 (9)	0.0005 (6)	−0.0013 (6)	−0.0035 (7)
C4	0.0166 (7)	0.0245 (8)	0.0271 (9)	0.0026 (6)	−0.0012 (6)	−0.0086 (7)
C5	0.0225 (8)	0.0176 (8)	0.0326 (10)	0.0027 (6)	−0.0056 (7)	−0.0015 (7)
C6	0.0209 (8)	0.0227 (8)	0.0253 (9)	−0.0012 (6)	−0.0028 (7)	0.0036 (7)
C7	0.0264 (8)	0.0320 (9)	0.0234 (9)	−0.0006 (7)	0.0050 (7)	−0.0050 (7)
C8	0.0166 (7)	0.0215 (8)	0.0210 (9)	−0.0056 (6)	0.0023 (6)	0.0008 (7)
C9	0.0225 (8)	0.0196 (8)	0.0176 (8)	−0.0022 (6)	0.0072 (6)	−0.0030 (6)
C10	0.0211 (8)	0.0195 (8)	0.0235 (9)	−0.0036 (6)	0.0029 (7)	−0.0005 (7)
C11	0.0174 (7)	0.0218 (8)	0.0188 (9)	0.0011 (6)	−0.0008 (6)	−0.0005 (7)
C12	0.0172 (7)	0.0213 (8)	0.0194 (8)	−0.0012 (6)	−0.0006 (6)	0.0026 (6)
C13	0.0239 (8)	0.0206 (8)	0.0189 (9)	0.0014 (6)	−0.0025 (7)	−0.0015 (7)
C14	0.0231 (8)	0.0293 (9)	0.0140 (8)	0.0059 (6)	0.0010 (6)	0.0024 (7)
C15	0.0231 (8)	0.0275 (9)	0.0211 (9)	−0.0013 (6)	0.0020 (7)	0.0063 (7)
C16	0.0228 (8)	0.0214 (8)	0.0217 (9)	−0.0023 (6)	0.0005 (7)	0.0014 (7)
C17	0.0242 (8)	0.0204 (8)	0.0185 (9)	0.0015 (6)	0.0038 (6)	−0.0006 (7)
C18	0.0164 (7)	0.0171 (7)	0.0230 (9)	0.0025 (6)	−0.0006 (6)	0.0016 (6)
C19	0.0208 (7)	0.0224 (8)	0.0249 (9)	−0.0005 (6)	0.0003 (7)	−0.0030 (7)
C20	0.0250 (8)	0.0346 (10)	0.0305 (10)	0.0012 (7)	0.0047 (7)	−0.0046 (8)
C21	0.0231 (9)	0.0382 (10)	0.0418 (12)	−0.0030 (7)	0.0100 (8)	0.0046 (9)
C22	0.0224 (8)	0.0237 (9)	0.0442 (12)	−0.0049 (6)	−0.0004 (8)	0.0019 (8)
C23	0.0209 (8)	0.0173 (8)	0.0332 (10)	0.0016 (6)	−0.0039 (7)	−0.0020 (7)

### Geometric parameters (Å, °)

**Table:**

Cl1—C12	1.7326 (15)	C8—C9	1.536 (2)
Cl2—C14	1.7370 (16)	C9—C17	1.527 (2)
O1—C8	1.2136 (19)	C9—H9	0.9800
O2—C10	1.3582 (18)	C10—C11	1.483 (2)
O2—C9	1.4313 (18)	C11—C16	1.394 (2)
O3—C10	1.1990 (18)	C11—C12	1.394 (2)
N1—C18	1.365 (2)	C12—C13	1.382 (2)
N1—N2	1.3660 (18)	C13—C14	1.377 (2)
N1—C17	1.4557 (19)	C13—H13	0.9300
N2—N3	1.3004 (19)	C14—C15	1.383 (2)
N3—C23	1.377 (2)	C15—C16	1.381 (2)
C1—C2	1.392 (2)	C15—H15	0.9300
C1—C6	1.402 (2)	C16—H16	0.9300
C1—C8	1.480 (2)	C17—H17A	0.9700
C2—C3	1.391 (2)	C17—H17B	0.9700
C2—H2	0.9300	C18—C19	1.394 (2)
C3—C4	1.400 (2)	C18—C23	1.398 (2)
C3—C7	1.500 (2)	C19—C20	1.373 (2)
C4—C5	1.383 (2)	C19—H19	0.9300
C4—H4	0.9300	C20—C21	1.414 (2)
C5—C6	1.377 (2)	C20—H20	0.9300
C5—H5	0.9300	C21—C22	1.360 (3)
C6—H6	0.9300	C21—H21	0.9300
C7—H7A	0.9600	C22—C23	1.403 (2)
C7—H7B	0.9600	C22—H22	0.9300
C7—H7C	0.9600		
			
C10—O2—C9	116.18 (11)	C16—C11—C12	118.02 (15)
C18—N1—N2	109.82 (12)	C16—C11—C10	119.32 (14)
C18—N1—C17	130.54 (13)	C12—C11—C10	122.67 (14)
N2—N1—C17	118.85 (13)	C13—C12—C11	121.03 (14)
N3—N2—N1	109.06 (13)	C13—C12—Cl1	117.22 (12)
N2—N3—C23	108.31 (13)	C11—C12—Cl1	121.69 (12)
C2—C1—C6	119.34 (14)	C14—C13—C12	119.16 (15)
C2—C1—C8	122.52 (14)	C14—C13—H13	120.4
C6—C1—C8	118.13 (14)	C12—C13—H13	120.4
C3—C2—C1	121.17 (14)	C13—C14—C15	121.67 (15)
C3—C2—H2	119.4	C13—C14—Cl2	118.56 (13)
C1—C2—H2	119.4	C15—C14—Cl2	119.77 (12)
C2—C3—C4	118.33 (15)	C16—C15—C14	118.29 (14)
C2—C3—C7	120.96 (14)	C16—C15—H15	120.9
C4—C3—C7	120.72 (14)	C14—C15—H15	120.9
C5—C4—C3	120.83 (15)	C15—C16—C11	121.81 (15)
C5—C4—H4	119.6	C15—C16—H16	119.1
C3—C4—H4	119.6	C11—C16—H16	119.1
C6—C5—C4	120.49 (15)	N1—C17—C9	114.27 (12)
C6—C5—H5	119.8	N1—C17—H17A	108.7
C4—C5—H5	119.8	C9—C17—H17A	108.7
C5—C6—C1	119.82 (15)	N1—C17—H17B	108.7
C5—C6—H6	120.1	C9—C17—H17B	108.7
C1—C6—H6	120.1	H17A—C17—H17B	107.6
C3—C7—H7A	109.5	N1—C18—C19	133.41 (14)
C3—C7—H7B	109.5	N1—C18—C23	104.14 (14)
H7A—C7—H7B	109.5	C19—C18—C23	122.41 (15)
C3—C7—H7C	109.5	C20—C19—C18	116.44 (15)
H7A—C7—H7C	109.5	C20—C19—H19	121.8
H7B—C7—H7C	109.5	C18—C19—H19	121.8
O1—C8—C1	122.46 (14)	C19—C20—C21	121.62 (17)
O1—C8—C9	119.18 (14)	C19—C20—H20	119.2
C1—C8—C9	118.34 (13)	C21—C20—H20	119.2
O2—C9—C17	106.50 (11)	C22—C21—C20	121.75 (16)
O2—C9—C8	109.41 (12)	C22—C21—H21	119.1
C17—C9—C8	112.67 (12)	C20—C21—H21	119.1
O2—C9—H9	109.4	C21—C22—C23	117.64 (15)
C17—C9—H9	109.4	C21—C22—H22	121.2
C8—C9—H9	109.4	C23—C22—H22	121.2
O3—C10—O2	123.50 (15)	N3—C23—C18	108.66 (14)
O3—C10—C11	126.53 (14)	N3—C23—C22	131.17 (14)
O2—C10—C11	109.96 (12)	C18—C23—C22	120.13 (15)
			
C18—N1—N2—N3	1.08 (16)	C10—C11—C12—Cl1	−2.4 (2)
C17—N1—N2—N3	171.98 (12)	C11—C12—C13—C14	−1.6 (2)
N1—N2—N3—C23	−1.23 (16)	Cl1—C12—C13—C14	−178.78 (11)
C6—C1—C2—C3	1.6 (2)	C12—C13—C14—C15	1.5 (2)
C8—C1—C2—C3	−177.02 (13)	C12—C13—C14—Cl2	−179.24 (11)
C1—C2—C3—C4	−0.6 (2)	C13—C14—C15—C16	−0.3 (2)
C1—C2—C3—C7	179.50 (13)	Cl2—C14—C15—C16	−179.58 (11)
C2—C3—C4—C5	−1.2 (2)	C14—C15—C16—C11	−0.8 (2)
C7—C3—C4—C5	178.73 (13)	C12—C11—C16—C15	0.7 (2)
C3—C4—C5—C6	1.9 (2)	C10—C11—C16—C15	−179.33 (13)
C4—C5—C6—C1	−0.9 (2)	C18—N1—C17—C9	−77.26 (19)
C2—C1—C6—C5	−0.9 (2)	N2—N1—C17—C9	114.04 (14)
C8—C1—C6—C5	177.82 (13)	O2—C9—C17—N1	74.44 (15)
C2—C1—C8—O1	162.53 (14)	C8—C9—C17—N1	−45.52 (17)
C6—C1—C8—O1	−16.1 (2)	N2—N1—C18—C19	177.33 (15)
C2—C1—C8—C9	−18.9 (2)	C17—N1—C18—C19	7.8 (3)
C6—C1—C8—C9	162.47 (13)	N2—N1—C18—C23	−0.46 (16)
C10—O2—C9—C17	162.00 (12)	C17—N1—C18—C23	−169.96 (14)
C10—O2—C9—C8	−75.96 (15)	N1—C18—C19—C20	−177.49 (16)
O1—C8—C9—O2	−7.23 (18)	C23—C18—C19—C20	0.0 (2)
C1—C8—C9—O2	174.12 (11)	C18—C19—C20—C21	0.5 (2)
O1—C8—C9—C17	111.04 (15)	C19—C20—C21—C22	−0.6 (3)
C1—C8—C9—C17	−67.61 (17)	C20—C21—C22—C23	0.2 (3)
C9—O2—C10—O3	−1.8 (2)	N2—N3—C23—C18	0.94 (17)
C9—O2—C10—C11	177.09 (12)	N2—N3—C23—C22	−176.75 (16)
O3—C10—C11—C16	130.70 (17)	N1—C18—C23—N3	−0.27 (17)
O2—C10—C11—C16	−48.10 (18)	C19—C18—C23—N3	−178.38 (13)
O3—C10—C11—C12	−49.3 (2)	N1—C18—C23—C22	177.72 (14)
O2—C10—C11—C12	131.91 (14)	C19—C18—C23—C22	−0.4 (2)
C16—C11—C12—C13	0.5 (2)	C21—C22—C23—N3	177.76 (16)
C10—C11—C12—C13	−179.47 (13)	C21—C22—C23—C18	0.3 (2)
C16—C11—C12—Cl1	177.60 (11)		

### Hydrogen-bond geometry (Å, °)

**Table:**

*D*—H···*A*	*D*—H	H···*A*	*D*···*A*	*D*—H···*A*
C15—H15···O1^i^	0.93	2.35	3.263 (2)	168
C4—H4···Cg1^ii^	0.93	2.86	3.4645 (19)	124

Symmetry codes: (i) −*x*+1, −*y*+2, −*z*; (ii) −*x*+1, *y*+1/2, −*z*+1/2.
